# Investigating Changes in Reward-Related Neural Correlates After PEERS Intervention in Adolescents With ASD: Preliminary Evidence of a “Precision Medicine” Approach

**DOI:** 10.3389/fpsyt.2021.742280

**Published:** 2021-11-03

**Authors:** Elizabeth Baker, Elina Veytsman, Tricia Choy, Jan Blacher, Katherine K. M. Stavropoulos

**Affiliations:** School of Education, University of California, Riverside, Riverside, CA, United States

**Keywords:** reward processing, PEERS intervention, autism, social motivation, precision medicine

## Abstract

**Background:** The Social Motivation Hypothesis proposes that individuals with autism spectrum disorder (ASD) experience social interactions as less rewarding than their neurotypical (TD) peers, which may lead to reduced social initiation. Existing studies of the brain's reward system in individuals with ASD report varied findings for anticipation of and response to social rewards. Given discrepant findings, the anticipation of and response to social rewards should be further evaluated, particularly in the context of intervention outcome. We hypothesized that individual characteristics may help predict neural changes from pre- to post-intervention.

**Methods:** Thirteen adolescents with ASD received the Program for the Education and Enrichment of Relational Skills (PEERS) intervention for 16 weeks; reward-related EEG was collected before and after intervention. Fourteen TD adolescents were tested at two timepoints but did not receive intervention. Event-related potentials were calculated to measure anticipation of (stimulus-preceding negativity; SPN) and response to (reward-related positivity; RewP) social and non-social rewards. Additionally, measures of social responsiveness, social skills, and intervention-engagement were collected. Group differences were analyzed as well as individual differences using prediction models.

**Result:** Parent-reported social responsiveness and social skills improved in adolescents with ASD after participation in PEERS. ASD adolescents displayed marginally decreased anticipation of social rewards at post-intervention compared to pre-intervention. Regression models demonstrated that older adolescents and those with *lower* parent-reported social motivation prior to participation in PEERS displayed marginally increased social reward anticipation (more robust SPN) from pre- to post-intervention. Participants who displayed more parent-reported social motivation before intervention and were more actively engaged in the PEERS intervention evidenced increased social reward processing (more robust RewP) from pre- to post-intervention.

**Conclusion:** Findings suggest that there may be differences in saliency between wanting/anticipating social rewards vs. liking/responding to social rewards in individuals with ASD. Our findings support the hypothesis that identification of individual differences may predict which adolescents are poised to benefit the most from particular interventions. As such, reported findings set the stage for the advancement of “precision medicine.” This investigation is a critical step forward in our ability to understand and predict individual response to interventions in individuals with ASD.

## Introduction

*There is a current lack of universally accepted terminology for describing autism* *(**1**)* *and as such, several terms are used in this paper to describe adolescents with autism. We used both person-first language and identity-first language in an effort to be inclusive of numerous current perspectives on appropriate terminology*.

### Autism and Social Motivation

Children with autism spectrum disorder (ASD) have reduced preferences toward social information compared to their neurotypical or typically developing (TD) peers (2, 3). The Social Motivation Hypothesis proposes that the brain's reward centers are related to early impairments in social attention due to social stimuli being less rewarding, thus setting a series of negative developmental consequences in motion (4). This may result in a reduction in social orienting, social interaction, and social skills—all of which may lead to broader deficits in social behaviors (4). Demonstration of the social motivation hypothesis often relies on the use of brain-based methods, including neural and neuropsychological markers of reward processing (5). Reward centers of the brain include mesolimbic dopamine system, comprised of the midbrain (via the ventral tegmental area) and striatum (via the nucleus accumbens) (6, 7).

### Social Motivation and Neural Response

Though some research suggests that children with ASD have less reward-related brain activity than their neurotypical peers in response to faces (8, 9), other work suggests that individuals with ASD evidence hypoactivity in the reward system in response to all stimulus types (10).

One way to approach mixed findings is by examining differences in reward-related brain activity by evaluating the difference between *anticipating* vs. *processing* rewards. Anticipation is linked to cues of reward and may become reinforced when the reward is more attractive or salient. Similarly, response to reward (i.e., reward processing) is enhanced if the reward is preferred but dampened if the reward is non-preferred. Anticipation of and response to rewards involve separate cognitive processes and both processes should be investigated in order to understand the entirety of how the reward system functions in individuals with and without ASD. Moreover, metrics of anticipation tend to be overlooked in paradigms designed to measure reward processing (11), which may contribute to mixed neural findings. A meta-analysis of functional magnetic resonance imaging (fMRI) studies examining anticipation of and response to rewards suggests that reward differences in ASD may apply to both social and non-social stimuli (12). Specifically, the caudate, nucleus accumbens, and anterior cingulate gyrus were hypoactive during anticipation of and in response to social and non-social rewards (12). These findings expand upon initial theories of disrupted reward systems more broadly.

Electroencephalographic (EEG) methods may serve to further elucidate the complexity of reward processing in ASD, as high temporal resolution is a notable feature and thus complements the high spatial resolution of fMRI. Additionally, EEG is a relatively inexpensive, non-invasive technique that is well-tolerated across the psychiatric spectrum. Using event-related potentials (ERPs), the stimulus-preceding negativity (SPN) component measures brain activity prior to stimulus presentation and may serve as a measure of anticipation. The reward-related positivity (RewP) ERP measures response to rewards and reflects the evaluation of rewards (i.e., determining if a reward is “liked” or “disliked”) by comparing losses to gains (13, 14). There is evidence to suggest that the SPN and RewP support the social motivation hypothesis, as children with ASD with less severe social impairments display larger reward anticipation (SPN) (15) and reward response (RewP) to faces (16).

### Behavioral Interventions for ASD

Behavioral interventions have been designed to improve social communication skills in ASD—by augmenting interactions with others and helping individuals with ASD form meaningful relationships; for reviews see (17, 18). The Program for the Education and Enrichment of Relational Skills (PEERS) intervention is a manualized, evidence-based group intervention designed to provide adolescents with ASD skills to both make and keep friends; see methods section for additional details (19–21). PEERS is efficacious in increasing social skills, frequency of social get-togethers, and friendships (20, 22).

### Objective Outcome Measures for Intervention

Objective measures, including brain-based measures, may identify factors that result in favorable intervention outcomes. To our knowledge, <10 studies have been published using measures of neural response as either an outcome measure or predictor of response to empirically supported behavioral intervention in individuals with ASD (16, 23–30). Of these studies, four used fMRI, and five used EEG methodology (16, 23, 24, 29, 30). Seven measured brain activity both before and after interventions (16, 24–27, 29, 30), five of which found increased brain activity in response to social stimuli (e.g., while viewing faces or in response to point-light displays of biological motion) (16, 25–27, 29). A majority of these investigations were done in children under 5 years, leaving much to be learned regarding adolescents' neural response to intervention.

As such, there is a pressing need for biomarkers that can detect meaningful intervention outcomes. Biomarkers may also address the heterogeneity of ASD through the identification of homogeneous subgroups of individuals based on biological factors. The N170, a neural measure of face processing and perception, is currently the only psychiatric biomarker for ASD approved by the Food and Drug Administration (31). It has been shown to be a sensitive measure of change due to the effects intervention while also identifying groups of individuals with ASD who have similar pathophysiology (23, 29, 31). Social difficulties in autism are underscored by aberrant processing of social information, as evidenced by a slower response (longer N170 latency) to faces compared to TDs (32–34), including in response to emotional faces (35). Given that the N170 is also closely associated with social communication challenges in ASD, it is a biomarker grounded in core ASD symptomatology.

### Use of Neural Response Before and After PEERS

Of the aforementioned papers using measures of neural response as an intervention outcome measure, two looked at brain activity before and after participation in PEERS. Van Hecke et al. measured resting state EEG before and after PEERS (24). The authors found that after participating in PEERS, teens with ASD displayed increased left-dominant gamma asymmetry, such that their brain activity appeared similar to that of neurotypical teens (24). Left-hemisphere dominance is associated with increased motivation and affect, while right-hemisphere dominance is associated with withdrawal and negative emotional style (36, 37). Additionally, Van Hecke et al. (24) found that after intervention, teens with ASD who (a) displayed fewer symptoms of ASD, (b) had more get-togethers with other adolescents during the intervention, and (c) displayed greater understanding of PEERS-specific concepts showed the greatest relative left-hemisphere dominant EEG activity in the gamma band. Therefore, it appears that individual characteristics seem related to the degree of left-dominant pattern of hemispheric asymmetry post-intervention.

In a second investigation of brain activity before and after PEERS (16), there was evidence of enhanced reward processing (as measured by the RewP) in teens with ASD after completion of PEERS. These findings suggest a malleability of social motivation in adolescents with ASD after social skills training. Additionally, the investigators found that adolescents with ASD who displayed less robust social reward processing prior to intervention made the most gains in social responsiveness, social skills, and PEERS-specific knowledge after intervention (16). That is, teens with ASD who displayed *less* response to social rewards prior to PEERS appeared to benefit the most from intervention. Thus, it appears critical to measure the contribution of unique individual factors to identify which individuals stand to benefit the most from intervention.

One such individual factor that remains unexplored is teen engagement in behavioral intervention. Motivation to participate in intervention, by way of active participation within sessions, may predispose adolescents to receive more benefits compared to those who are less engaged. PEERS was originally validated in children and teens ages 11–16 years (22), a developmental period from late childhood through adolescence characterized by increased social demands (33). As such, age should be considered as a potential moderator to the effects of intervention. Age is also relevant in brain-based studies of reward processing, as younger individuals (e.g., early adolescents) with ASD appear to show greater variability in striatal activation during social reward tasks compared to older individuals with ASD, which may contribute to differences in anticipation vs. response processes in ASD (12).

### Current Study

The current study, which is a preliminary model of using a “precision medicine” approach to intervention, was designed to answer the following questions:

1. How does reward-related brain activity, both anticipation (SPN) and processing (RewP), to social and non-social stimuli change from pre- to post- PEERS intervention in a sample of adolescents with ASD?
2. How does brain activity related to anticipation of and response to social and non-social rewards differ across time between adolescents with ASD receiving PEERS vs. typically developing (TD) adolescents not receiving PEERS?
3. Does change in reward-related brain activity before and after intervention relate to individual factors? That is, can individual change in reward anticipation and processing from pre- to post- PEERS intervention be predicted by individual characteristics (e.g., age, social skills)?

To our knowledge, this is the first study to: (A) measure electrophysiological correlates of both anticipation of and response to social and non-social stimuli in teens with ASD before and after participation in PEERS, and (B) compare brain activity of teens with ASD before and after PEERS to brain activity of TD teens across time. Exploratory analyses on the N170 were performed after visual inspection of the ERP data; see Methods for details.

## Methods

### Participants

Participants included 13 adolescents with ASD and 14 sex-, age-, IQ-, and race-matched TD adolescents; see Table 1. A total of 17 ASD participants were initially enrolled in the study. However, four dropped out for reasons including: difficulty with transportation, psychiatric hospitalization, and the adolescent no longer wanting to attend sessions. Thus, 13 ASD participants were included in the final sample. The 14 TD participants were not enrolled in the PEERS intervention and instead were seen at two timepoints, 16 weeks apart. Though the sample size is modest, a majority of participants in the current study identified as Latinx. Much intervention research is carried out with White, monolingual English-speakers. This is one of the first studies to investigate the effect of PEERS in a diverse sample in which the intervention was carried out in a language-inclusive environment in both English and Spanish, see below.

**Table 1:** Descriptive characteristics of the autism spectrum disorder (ASD) and neurotypical (TD) groups at Time 1.

**Characteristics**	**ASD**	**TD**
	***n* = 13**	***n* = 14**
Sex	10 male, 3 female	12 male, 2 female
Age [M (SD), Range]	14.17 (2.09), 11.3–17.1	13.22 (1.63), 11.1–17.1
IQ, M (SD), Range	99.54 (15.62), 77–129	106.14 (15.49), 79–131
**Race (** ***n*** **)**		
White *n*	3	4
Latinx *n*	9	8
Mixed race/other *n*	1	2
**Maternal education level (** ***n*** **)**		
Less than college	10	5
College and above	3	9
**Household income (** ***n*** **)**		
Up to $50,000	4	4
$50, 001–100,000	5	4
Over $100,001	4	5
Missing data	–	1

*The ASD and TD samples are well-matched on sex, age, IQ, race, and household income. However, we note that maternal education is lower in the ASD group compared to the TD group*.

Flyers with study details were posted at community centers and events. Interested families with adolescents between the ages of 11–18 years were contacted via phone or email. Exclusionary criteria for the ASD and TD groups included: an IQ below 70, history of seizures/epilepsy, history of brain injury/disease, and a diagnosis of intellectual disability. Commonly co-occurring disorders were not exclusionary in the ASD group, though a history of serious psychiatric illness (e.g., schizophrenia, bipolar disorder) or a recent (within 6 months) psychiatric hospitalization was exclusionary. Additional exclusionary criteria for the TD group included a psychiatric diagnosis of any kind and immediate family history of ASD.

All participants in the ASD group had diagnosis confirmed with the Autism Diagnostic Observation Schedule, 2nd edition (ADOS-2) (38). The ADOS-2 was performed by research-reliable graduate students who had at least 5 years of experience working with individuals with ASD. ASD adolescents needed to have English as a primary language to be included in the intervention. Parents could speak either English or Spanish as parent groups were delivered in a bilingual format. A third timepoint set for 4 months later was scheduled to measure lasting impacts of intervention; however, COVID-19 prevented participants from returning to the lab to complete the EEG follow-up visit. This study was approved by the Institutional Review Board at the University of California, Riverside. Caregivers provided informed consent, and adolescents provided assent.

### Procedures, Assessments, and Questionnaires

Cognitive abilities were tested using the 2-subtest Wechsler Abbreviated Scales of Intelligence, 2nd edition (WASI-II) (39). Composite scores were combined to create a full-scale IQ-2 (FSIQ-2). For adolescents with ASD, diagnosis was confirmed using the ADOS-2 (38). ADOS-2 consists of five modules based upon the individual's language ability and age. In this study, Modules 3 and 4 were used for participants with ASD. Willingness to participate the intervention was assessed in ASD participants using the Mental Status Checklist (21). These measures were used to confirm eligibility and therefore were not repeated.

Caregivers completed the Social Responsiveness Scale, Second Edition (SRS-2) (40), and the Social Skills Improvement System (SSIS) (41) before the intervention began (Time 1) and immediately after intervention completion (Time 2). Times 1 and 2 were ~4 months apart, as the duration of the PEERS intervention is 16 weeks. The same EEG task was completed by adolescents in both groups at Time 1 and Time 2.

The SRS-2 is a standardized 65-item parent-report rating scale used to assess the severity of autism symptoms and social responsiveness in children ages 4 to 18 (40). A Total Score is calculated from five subscales: Social Awareness, Social Cognition, Social Communication, Social Motivation, and Restricted Interests and Repetitive Behavior.

The SSIS is a standardized 79-item parent-report measure of social and behavioral functioning for children ages 3 to 18 (41). The measure is designed to assess treatment-related changes in social skills (subscale: Social Skills) and problem behaviors (subscale: Problem Behaviors).

Teen engagement in intervention sessions was measured by tallying the number of times adolescents actively participated (e.g., asking questions, making comments, reporting on homework assignments). The tallies were recorded by the interventionist during active sessions. A sum of participation across 16 sessions was calculated. This metric is referred to below as “Teen Participation.” See Table 2 for SRS-2, SSIS, and Teen Participation means.

**Table 2:** Mean scores on behavioral measures in TD and ASD participants at Time 1 and Time 2.

	**TD**		**ASD**	
	**Time 1**	**Time 2**	**Time 1**	**Time 2**
	**M (SD)**	**M (SD)**	**M (SD)**	**M (SD)**
SRS-2 total T-score	45.29 (6.33)	44.07 (6.38)	74.85 (12.84)	68.85 (15.06)
SRS-2 social motivation T-score	49.21 (8.83)	47.43 (9.23)	75.15 (14.97)	70.77 (17.76)
SSIS social skills standard score	105.64 (11.89)	105.21 (12.59)	81.62 (19.19)	87.85 (19.05)
Teen participation	—		256.31 (91.38), range: 165–469	

*Higher SRS-2 scores indicate greater severity, while lower SSIS scores indicate greater severity*.

### Social Skills Intervention: PEERS

PEERS is a 16-week, outpatient, manualized intervention to help adolescents make and keep friends (19–22, 42). The PEERS intervention consists of weekly, 1.5-h group sessions for parents and teens. Parent groups are conducted in a separate room from adolescent groups. Adolescent group sessions focused on teaching social skills specific to making and keeping friends and handling peer conflict and rejection. Skills were taught using didactic instruction which included role-play demonstrations, behavioral rehearsal activities with reinforcement and corrective feedback, and weekly homework assignments (43). Parent group sessions were provided in a bilingual format. All written parent materials were available in Spanish and English. Each group was led by a trained interventionist. All procedures were supervised by a licensed psychologist.

### EEG

#### EEG Task

The EEG task was completed by ASD and TD participants at Time 1/pre-intervention and Time 2/post-intervention. The EEG task included two blocks of 50 trials, each comprised of one of two conditions (social or non-social). In both blocks, at the beginning of each trial, a fixation cross appeared on the screen for 500 milliseconds (ms). After the fixation cross, two boxes, each containing a question mark, were displayed. Participants were instructed to indicate their guess via a button pad regarding whether the left or right stimulus was “correct.” The boxes were displayed until participants made a choice—up to 3,000 ms. If participants did not make a choice after 3,000 ms the trial ended and the next trial began. After participants indicated their choice, an arrow appeared pointing in the direction of the box they picked for 3,000 ms. After 3,000 ms, feedback appeared to indicate if the participant guessed correctly or incorrectly (displayed for 1,000 ms).

In the social condition, feedback was an image of a smiling face from the “NimStim” database (44) surrounded by intact Oreo cookies for correct answers or an image of a frowning face surrounded by crossed out Oreo cookies for incorrect answers. In the non-social condition, feedback was an image of an upward arrow surrounded by Oreo cookies for correct answers or an image of a downward arrow surrounded by crossed out Oreo cookies for incorrect answers. Arrow stimuli were composed of scrambled face elements from the social condition. A computer program predetermined correct vs. incorrect answers in semi-random order such that participants got 50% “correct” and 50% “incorrect,” with no more than three of the same feedback in a row. Each trial was marked to be correct vs. incorrect regardless of the participant's response.

**Figure 1:**
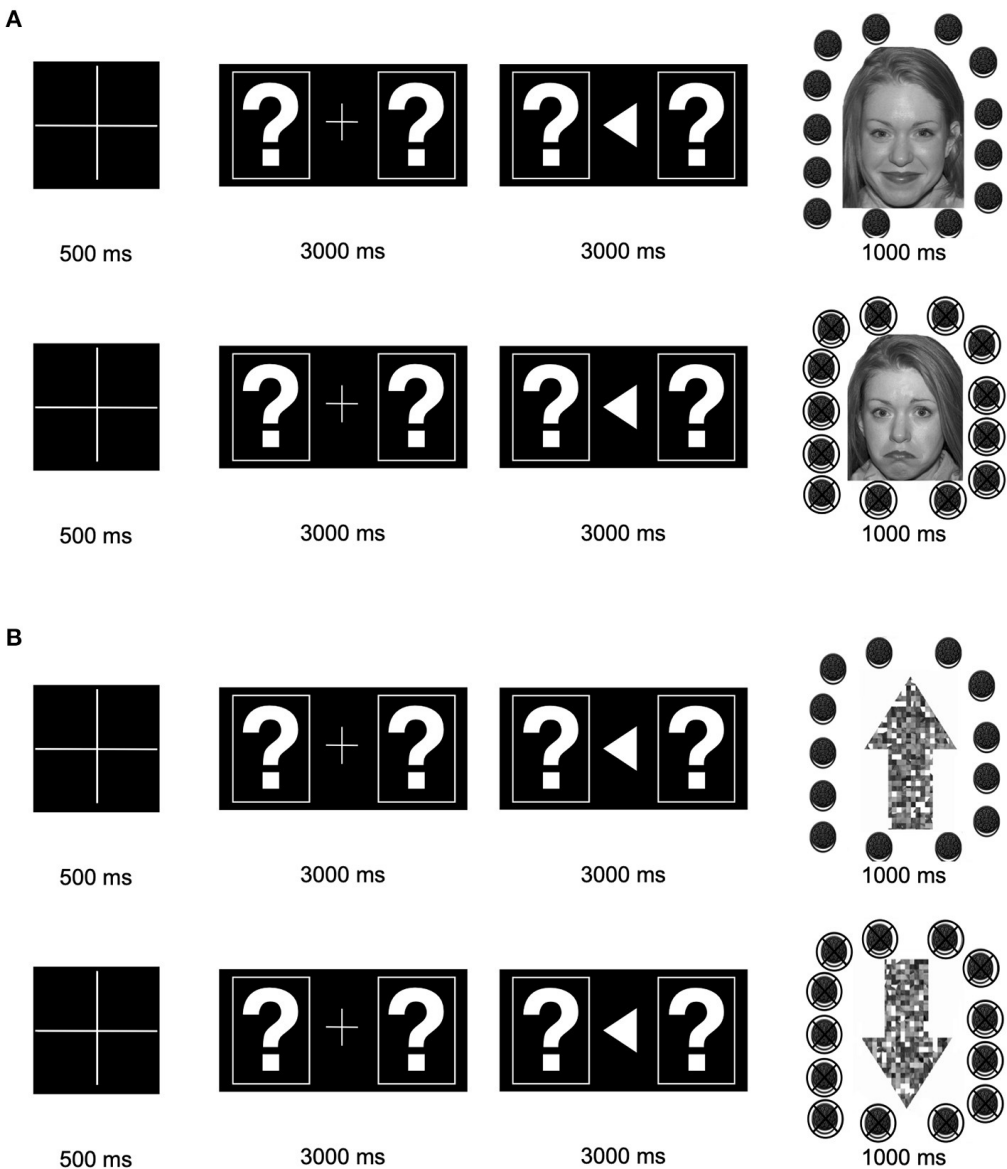
Stimulus presentation: **(A)** Stimuli and presentation timing for the social condition. **(B)** Stimuli and presentation timing for the non-social condition. Correct feedback is shown on top (intact Oreos); incorrect feedback is shown on the bottom (crossed-out Oreos).

Participants were verbally told that the reward for correct answers was Oreo cookies (or an equivalent snack). Importantly, in both the social and non-social feedback trials, the face/arrow information was incidental: it was not necessary for the participant to determine whether their response was correct. Participants were told that correct vs. incorrect responses were signaled by whether the Oreo cookies were intact or crossed out. Whether individuals viewed the social vs. non-social block first was counterbalanced. See Figure 1.

#### EEG Recording and Processing

Participants wore a standard, fitted cap (Brain Products ActiCap) with 32 silver/silver-chloride (Ag/AgCl) electrodes placed according to the extended international 10–20 system. Continuous EEG was recorded using Brain Vision Recorder with a reference electrode at Cz and re-referenced offline to average activity at left and right mastoids. Electrode resistance was kept under 50 kOhms. Continuous EEG was amplified with a directly coupled high pass filter (DC) and notch filter (60 Hz). The signal was digitized at a rate of 500 samples per second. Eye movement artifacts and blinks were monitored via horizontal electrooculogram (EOG) placed at the outer canthi of each eye and vertical EOG placed above and below the left eye.

Trials with no behavioral response, or containing electrophysiological artifacts, were excluded. Artifacts were removed via a four-step process. Data were visually inspected for drift exceeding +/−200 mV in all electrodes, high frequency noise visible in electrodes larger than 100 mV, and flatlined data. Following inspection, data were epoched and eyeblink artifacts were identified using independent component analysis (ICA). Individual components were inspected alongside epoched data, and blink components were removed. To remove additional artifacts, we utilized a moving window peak-to-peak procedure in ERPlab (45), with a 200 ms moving window, a 100 ms window step, and a 150 mV voltage threshold.

##### SPN

**Figure 2:**
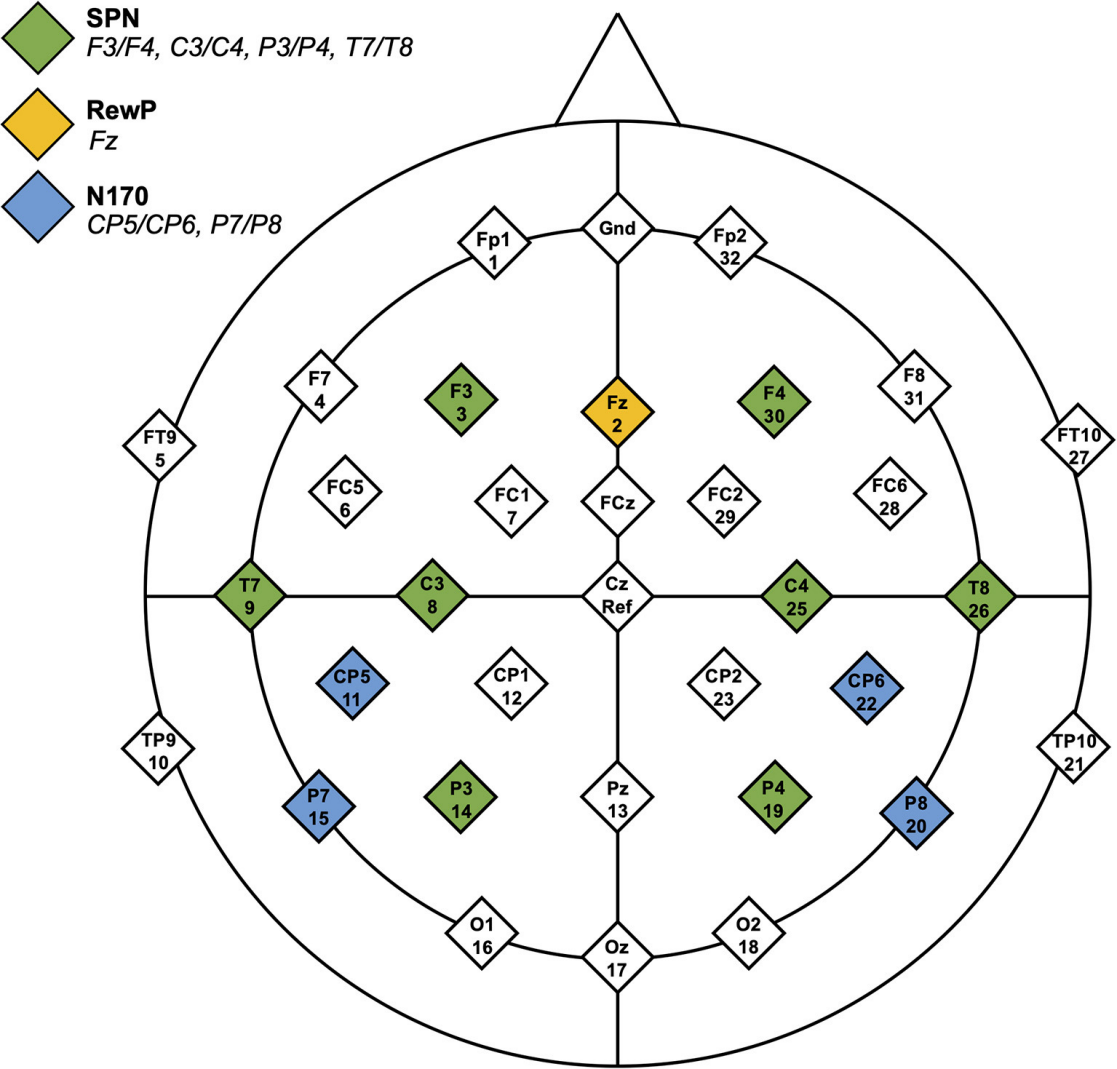
Headmap of electrode positions displaying regions of interest for the SPN, RewP, and N170 components.

Baseline was −3,200 to −3,000 ms, and the data were epoched from −3,200 to 100 ms (time-locked to the onset of feedback stimuli). SPN mean amplitude between −210 and −10 ms was calculated for social and non-social conditions. Electrode locations included F3/F4, C3/C4, P3/P4, and T7/T8. See Figure 2 for electrode locations.

##### RewP

Baseline was set to −100 to 0 ms, and the data were epoched from −100 to 800 ms. RewP mean amplitude was calculated for each condition from the frontocentral electrode, Fz (46, 47). For both conditions (face, arrow) and both feedback types (correct, incorrect), mean brain activity was calculated between 275 and 425 ms after feedback onset. The RewP was defined as a difference wave where brain activity in response to “incorrect” feedback was subtracted from brain activity in response to “correct” feedback.

##### N170

Upon visual inspection of grand average EEG data files, a negative-going deflection was observed after stimulus presentation, particularly in the social condition. Though the EEG stimuli in the current investigation were designed to elicit reward anticipation and response, exploratory analyses of the N170 are included. Only social and non-social trials with correct feedback (i.e., smiling faces and upwards-facing arrows) were analyzed. Incorrect trials were excluded from N170 analyses to eliminate confounds related to processing negative emotional valences (48) (i.e., frowning faces). The baseline period was set to −100 to 0 ms and data were epoched from −100 to 800 ms. Peak amplitude and latency were calculated between 150 and 250 ms in CP5/CP6 and P7/P8 electrodes (33, 49).

#### EEG Data Retention

Of the 13 ASD participants included in this investigation, 12 participants provided a minimum of 10 trials in the social and non-social conditions at Time 1 and Time 2. Thus, 12 ASD participants were included in analyses of the SPN, RewP, and N170.

All 14 TD were included in RewP and N170 analyses, as each participant provided a minimum of 10 trials per condition at each timepoint. For SPN analyses, four TD participants did not provide the necessary 10 trials per condition at both timepoints, resulting in a total of 10 TD participants included in SPN analyses.

### Statistical Analyses

All analyses were conducted using SPSS Version 27 (2020). Repeated-measures analyses of variance (ANOVAs) were conducted to test the effects of condition (social, non-social), time (pre-, post-intervention), and group (ASD, TD) on SPN mean amplitude, RewP mean amplitude, and N170 peak amplitude and latency. ANOVAs were conducted with Age at Time 1 as a covariate.

Repeated-measures ANOVAs were conducted to test the effects of group and time on behavioral measures of interest (i.e., SRS-2, SSIS, and Teen Engagement). Pearson correlations were conducted to test which pre-intervention measures were significantly associated with change in ERPs after intervention in the ASD group. Change in SPN and RewP was calculated as a difference score by subtracting pre-intervention mean amplitudes from post-intervention mean amplitudes within social and nonsocial conditions, respectively. Though there are some methodological concerns surrounding the use of change scores (e.g., reliability), they were used in this investigation due to their robustness against non-randomized designs, particularly when change scores are included as a dependent variable in regression analyses (50). Pearson correlations between behavioral variables of interest at Time 1 (pre-intervention) and ERP difference scores in the ASD group from Time 1 (pre-intervention) to Time 2 (post-intervention) were conducted to determine which variables to include in linear regression models. Finally, separate linear regressions were conducted in the ASD group based on the results of the correlations between behavioral measures at Time 1 and changes in brain activity from Time 1 to Time 2. The number of independent variables included in a multivariate regression is often determined using a 20:1 ratio, such that there should be 20 subjects for each independent variable (51, 52). Given the small sample size in this investigation, separate univariate regressions were conducted as to not violate basic principles. No prediction models including the N170 were conducted, as these analyses were exploratory.

## Results

### ERP

#### SPN

Prior to running ANOVAs to test the effect of intervention and group on SPN amplitude, differences by hemisphere and electrode position were conducted using a 2 (hemisphere: left, right) × 2 (time) × 4 electrode position (Frontal, Central, Parietal, Temporal) ANOVA. No significant main effects or interactions were found. As such, ANOVAs were collapsed across hemisphere and electrode position, similar to prior investigations using the same ERP paradigm (9, 53). Note that some of these values are at the margin of statistical significance; analyses were reported for hypothesis-generating purposes and to inform future research.

**Figure 3:**
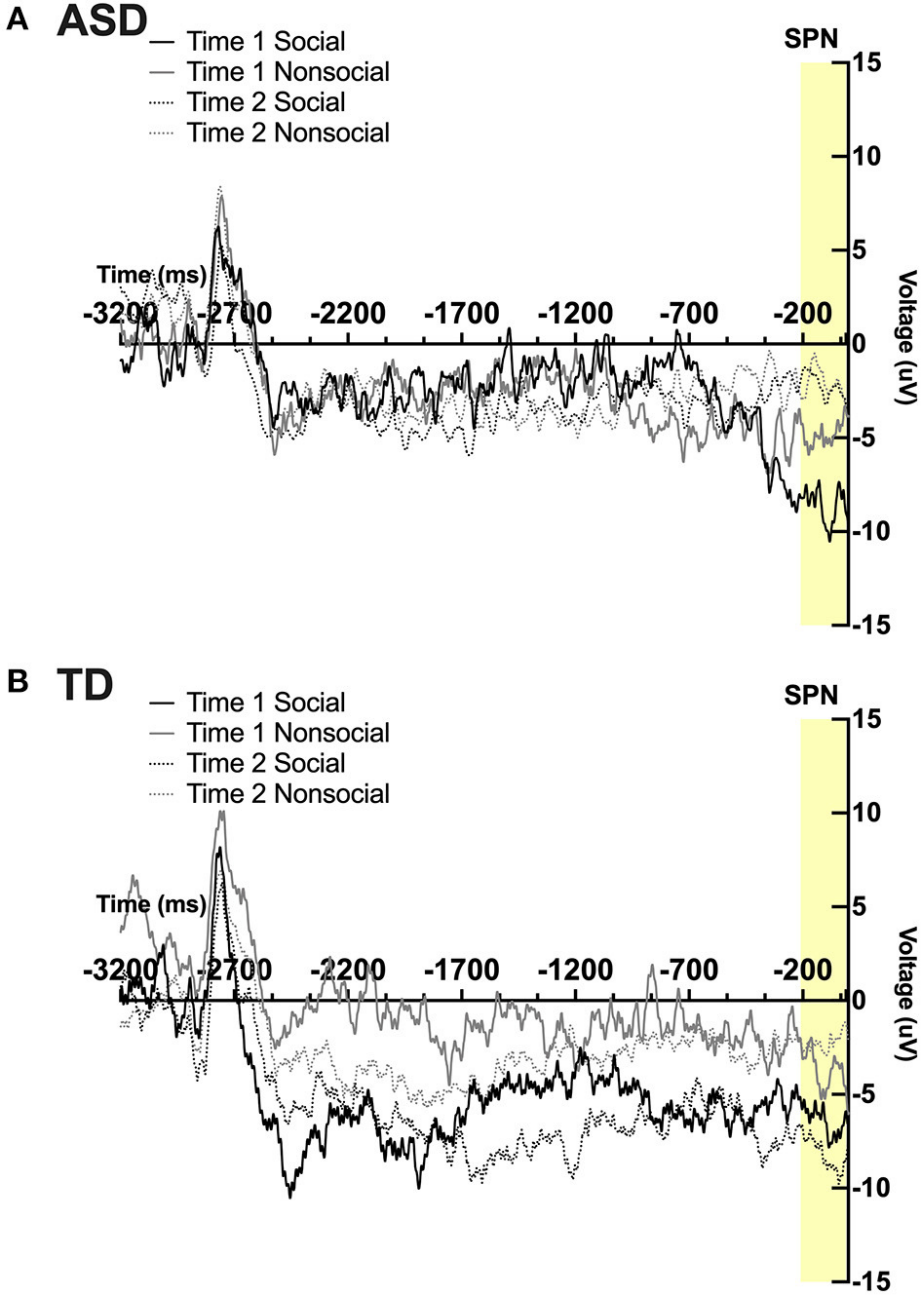
Grand average waveforms in the social and non-social conditions at Time 1 and Time 2 from the Stimulus Preceding Negativity (SPN) in **(A)** ASD participants and **(B)** TD participants.

A significant 2-way interaction was found between time and condition; *F*_(1,19)_ = 6.07; *p* = 0.02, η_*p*_^2^ = 0.24. A marginally significant 3-way interaction was found between time, condition, and group; *F*_(1,19)_ = 4.09, *p* = 0.057, η_*p*_^2^ = 0.18. Pairwise comparisons revealed a marginally significant effect of time in the ASD group, such that participants had marginally smaller SPN magnitude in the social condition at post-intervention compared to pre-intervention; *F*_(1,19)_ = 4.14, *p* = 0.056. Pairwise comparisons also revealed a marginal effect of condition at Time 2 in the TD group such that TD participants displayed a marginally more robust SPN to faces vs. arrows at time 2; *F*_(1,19)_ = 3.34, *p* = 0.083. No other main effects or interactions were observed. See Figures 3A,B.

#### RewP

**Figure 4:**
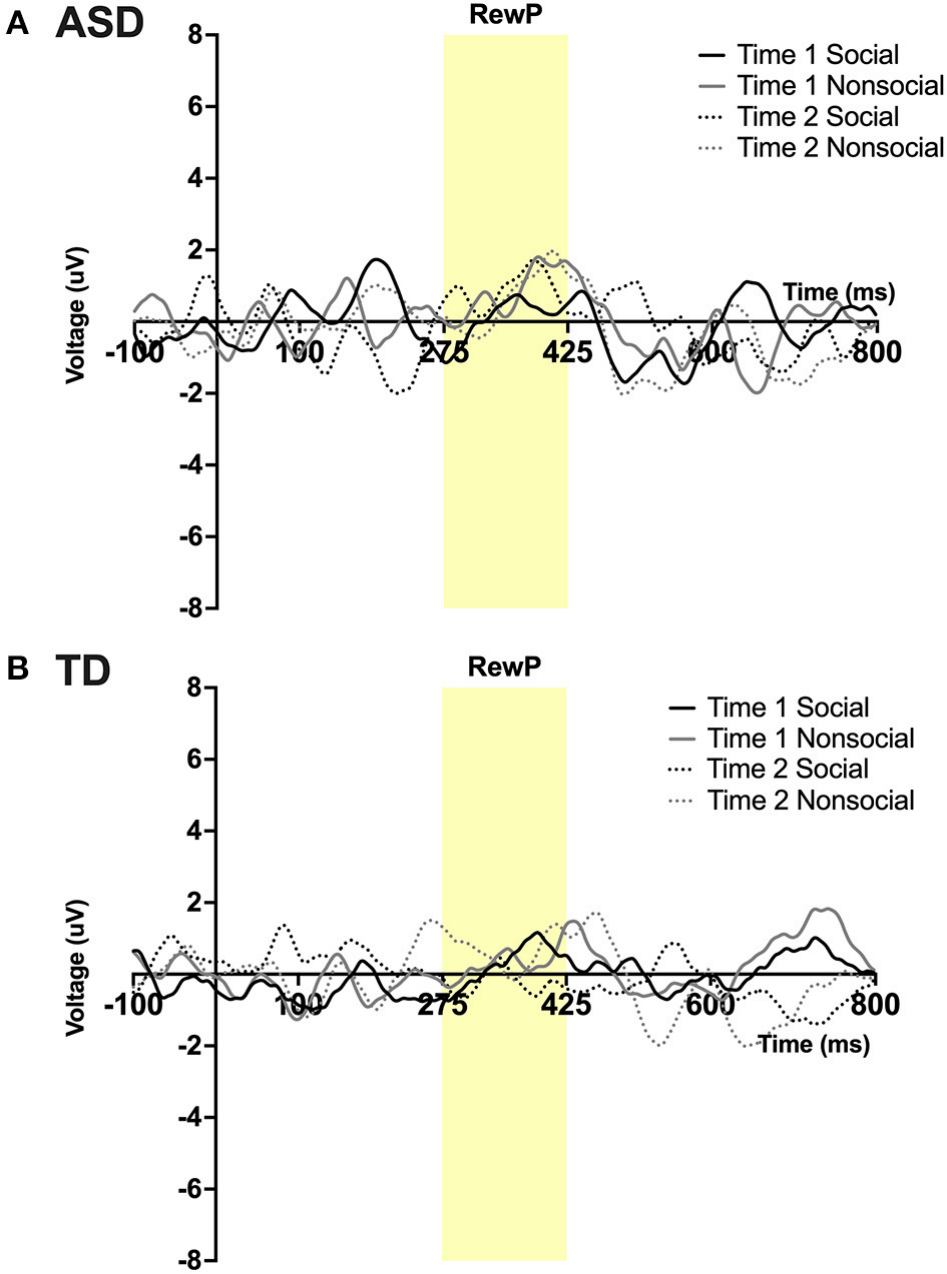
Grand average waveforms in the social and non-social conditions at Time 1 and Time 2 from Reward Positivity (RewP) ERP in **(A)** ASD participants and **(B)** TD participants. Note that for this figure, ERPs were filtered using a 25 Hz low-pass filter.

A main effect of condition was found; *F*_(1,23)_ = 5.15, *p* = 0 .03, η_*p*_^2^ = 0.18 such that all participants, regardless of time, had a more robust RewP mean amplitude in response to social vs. non-social stimuli. No other main effects or interactions were observed. See Figures 4A,B.

#### Exploratory Analysis: N170 Peak Amplitude

**Figure 5:**
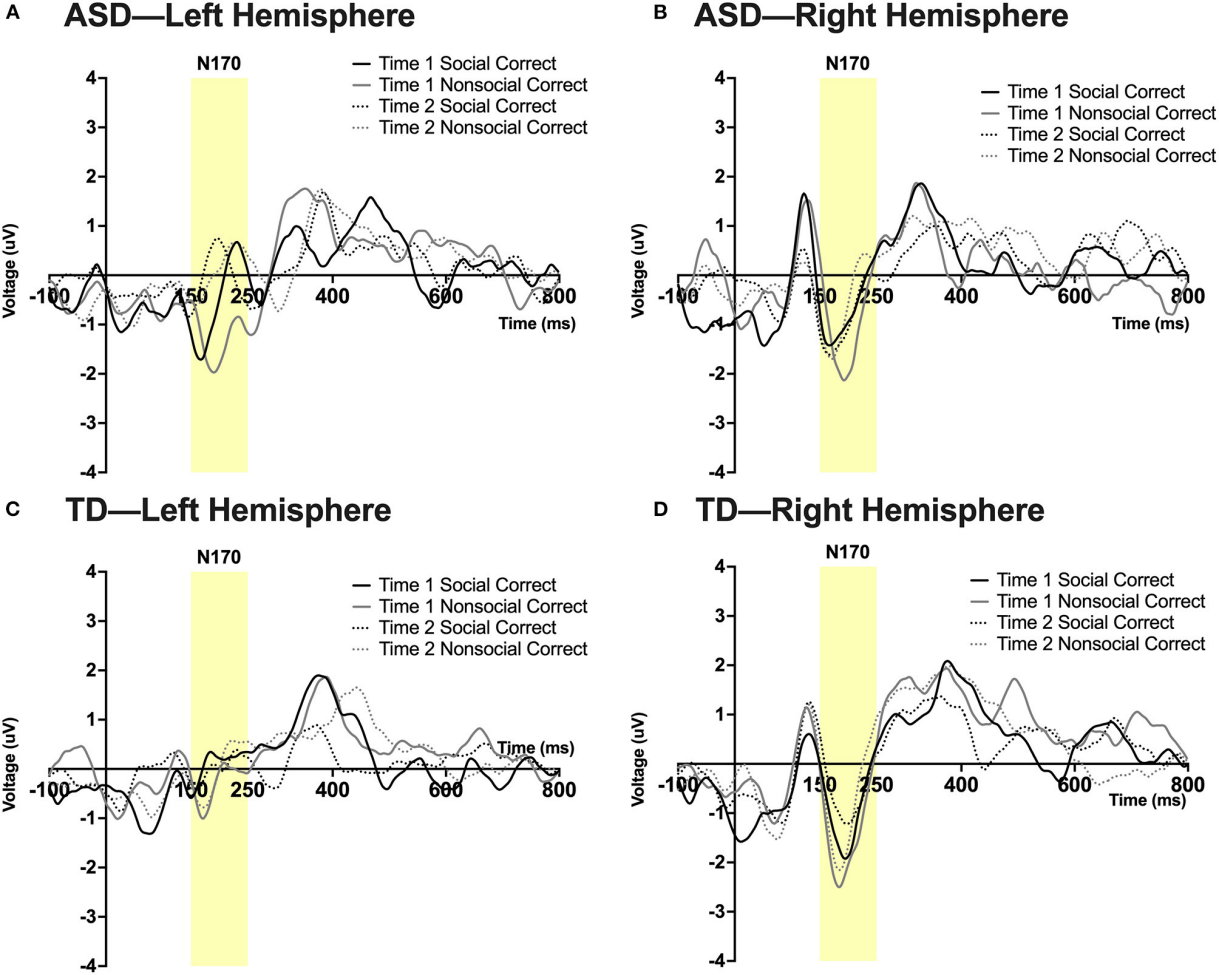
Grand average waveforms in the social and non-social conditions at Time 1 and Time 2 for the N170 ERP in **(A)** ASD participants in the left hemisphere, **(B)** ASD participants in the right hemisphere, **(C)** TD participants in the left hemisphere, and **(D)** TD participants in the right hemisphere. Note that for this figure, ERPs were filtered using a 25 Hz low-pass filter.

See note above; some of these values are at the margin of statistical significance. A significant 3-way interaction was found between time, hemisphere, and group; *F*_(1,23)_ = 13.35, *p* = 0.045, η_*p*_^2^ = 0.16. A 4-way interaction was found between time, condition, hemisphere, and group; *F*_(1,23)_ = 14.19, *p* = 0.027, η_*p*_^2^ = 0.195. Pairwise comparisons revealed that in the right hemisphere at Time 1, the ASD group had a more robust N170 than the TD group in the social condition; *F*_(1,23)_ = 5.14, *p* = 0.033. In the ASD group there was a marginal effect of time such that in the right hemisphere there was a more robust N170 in the social condition at Time 2 (post-intervention) compared to Time 1 (pre-intervention); *F*_(1,23)_ = 3.99, *p* = 0.058. In the TD group at Time 1, a more robust N170 was found in the non-social compared to the social condition in both left [*F*_(1,23)_ = 6.08, *p* = 0.022] and right hemispheres [*F*_(1,23)_ = 4.57, *p* = 0.043]. Additionally, a marginally significant effect of hemisphere was observed in the TD group at Time 1 in the social condition such that a more robust N170 was observed in the right vs. left hemisphere; *F*_(1,23)_ = 3.86, *p* = 0.062. See Figure 5.

#### N170 Latency

A main effect of hemisphere was observed, *F*_(1,23)_ = 5.802, *p* = 0.024, η_*p*_^2^ = 0.20, such that the left hemisphere had a shorter N170 latency than the right hemisphere. No other main effects or interactions were observed.

### Behavioral Results: Repeated Measures ANOVA

Three 2 (group) × 2 (time) repeated measures ANOVAs were conducted to measure changes in SRS-2 Total score, SRS-2 Social Motivation, and SSIS Social Skills from Time 1 to Time 2. For the SRS-2 Total score, there was a main effect of time; *F*_(1,25)_ = 9.66, *p* < 0.01, η_*p*_^2^ = 0.28; and a significant interaction between time and group; *F*_(1,23)_ = 4.25, *p* = 0.05, η_*p*_^2^ = 0.15. Pairwise comparisons revealed ASD participants had significantly higher SRS-2 Total scores at Time 1 [*F*_(1,25)_ = 58.94, *p* < 0.01, η_*p*_^2^ = 0.70] and Time 2 [*F*_(1,25)_ = 31.84, *p* < 0.01, η_*p*_^2^ = 0.56] compared to TD participants. ASD SRS-2 Total scores decreased from Time 1 to Time 2; *F*_(1,25)_ = 12.88, *p* < 0.01, η_*p*_^2^ = 0.34, while TD scores remained the same across time, *F*_(1,25)_ = 0.59, *p* = 0.49. A main effect of group was observed for the SRS-2 Social Motivation subscale [*F*_(1,25)_ = 27.26, *p* < 0.001, η_*p*_^2^ = 0.52] and SSIS Social Skills subscale, [*F*_(1,25)_ = 12.88, *p* < 0.01, η_*p*_^2^ = 0.34], such that TDs had lower Social Motivation T-scores and higher Social Skills Standard Scores than ASD participants, regardless of time. Note that for the SRS-2, lower scores indicate fewer symptoms of ASD, whereas on the SSIS, higher sores indicate fewer social skills impairments. Refer to Table 2 for mean values.

### ERP and Behavior: Correlations and Linear Regressions

#### Correlations

Note that some of these values are at the margin of statistical significance. The SPN social condition mean amplitude change was marginally correlated with pre-intervention age (*r* = −0.56, *p* = 0.059) and pre-intervention SRS-2 Social Motivation scores (*r* = −0.57, *p* = 0.055). Thus, increased magnitude of the SPN from Time 1 to Time 2 (note that the SPN more negative change scores reflect more robust reward anticipation) was correlated with older ages and worse social motivation prior to the start of intervention. Two additional correlations with the SPN social condition mean amplitude change trended toward significance. SPN mean amplitude change was negatively correlated with SRS-2 Total (*r* = −0.53, *p* = 0.079) and positively correlated with SSIS Social Skills (*r* = 0.54, *p* = 0.069).

The RewP social condition mean amplitude change was negatively correlated with SRS-2 Social Motivation scores pre-intervention (*r* = −0.67, *p* = 0.02), such that an increased reward response to social stimuli was correlated with better social motivation scores before the start of intervention. RewP social condition difference score was positively correlated with Teen Participation (*r* = 0.70, *p* = 0.01), such that increased reward response to social stimuli from Time 1 to Time 2 was correlated with more intervention engagement. See Table 3 for a summary of correlation and linear regression results.

**Table 3:** Results of correlations and linear regressions in the ASD Group only.

	**Correlation**		**Linear regression**				
	***r***	***p***	***B***	**SE B**	**β**	***t***	***p***
**SPN social condition change**							
Age T 1	−0.56	0.059	−3.27	1.53	−0.56	−2.133	0.059
SRS-2 social motivation T 1	−0.57	0.055	−0.484	0.22	−0.57	−2.17	0.055
SRS-2 total T 1	−0.53	0.079	–	–	–	–	–
SSIS social skills T 1	0.54	0.069	–	–	–	–	–
**RewP social condition change**							
SRS-2 social motivation T 1	−0.67	0.02	−0.32	0.11	−0.67	−2.85	0.02
Teen participation	0.70	0.01	0.05	0.02	0.70	3.10	0.01

*SPN social condition change and RewP social condition change are each outcome variables; all regressions were run separately*.

#### Linear Regressions

As stated above, some of these values are at the margin of statistical significance. Two linear regressions were conducted to test if age at the start of intervention and pre-intervention SRS-2 Social Motivation scores predicted change in SPN social condition mean amplitude. Thirty-two percent of the variance of the change in anticipation of social reward was accounted for by SRS-2 Social Motivation pre-intervention scores, β = −0.57*; F*_(1,10)_ = 4.71, *p* = 0.055. Thirty-one percent of the variance in change in anticipation of social reward was accounted for by age at the start of intervention, β = −0.56*; F*_(1,10)_ = 4.55, *p* = 0.059.

Two linear regressions were conducted in the ASD group to test if pre-intervention SRS-2 Social Motivation scores and Teen Participation predicted change in RewP social condition mean amplitude. Results revealed that 44.9% of the variance of the change in social reward responsivity (RewP mean amplitude in response to faces) was accounted for by SRS-2 Social Motivation pre-intervention scores, β = −0.67*; F*_(1,10)_ = 8.14, *p* = 0.02. Similarly, 49% of the variance of the change in social reward responsivity was accounted for by Teen Participation, β = 0.70; *F*_(1,10)_ = 9.60, *p* = 0.01.

## Discussion

Social behaviors were improved in adolescents with ASD in the areas of social responsiveness and social skills, such that a reduction in autism symptomatology was observed after participation in PEERS. In addition to behavioral improvements, changes in neural correlates of reward were detected. The primary aim of this study was to investigate anticipation of and response to reward-related brain activity before and after completion of PEERS and to examine the ways in which individual factors impacted outcomes. As such, this preliminary study is one of the first to examine reward-related brain activity before and after intervention with a group of teens with ASD. Additionally, this investigation included a majority Latinx sample, a historically underrepresented group. The inclusion of minority groups in intervention and in measures of neural response advances the representation of such groups and improves generalizability of findings.

### Anticipation

Participants with ASD displayed marginally less anticipation (less robust SPN) to social rewards at post-intervention compared to pre-intervention. Though contrary to our hypotheses, it is possible that increased comfort and familiarity with social situations may explain these findings. That is, increased familiarity and experiences in social settings and/or in social interactions may have dampened anticipation of social information, as social behaviors became routine throughout the course of intervention. In contrast, TD participants did not evidence differences in reward anticipation across time. However, marginal differences between social and non-social conditions were observed at Time 2 such that TD adolescents evidenced more anticipatory brain activity in response to social vs. non-social stimuli. Our findings suggest that participation in PEERS leads to changes in anticipation of social stimuli for adolescents with ASD, whereas time does not lead to equivalent changes for TD adolescents.

Individual variability of change in neural correlates of social anticipation from pre- to post-intervention was predicted by age and parent-reported social motivation at the beginning of the intervention. Older adolescents and those with *less* reported social motivation prior to PEERS displayed increased neural anticipation for faces from pre- to post-intervention. It will be important for future research to explore potential effects of age on PEERS efficacy, as the intervention is inclusive of a large age range. Our finding that teens with less social motivation prior to PEERS displayed increased social reward anticipation after PEERS is a critical step forward in our ability to understand why some participants may benefit more from intervention than others.

### Processing

In all participants, response to rewards was greater (more robust RewP) to social compared to non-social stimuli. Though previous work has reported hypoactivation in reward-related brain areas to social stimuli (54), findings in the current study provide an alternative account. It is possible that social deficits unique to ASD may not be reliably detected at the neural level in all children/adolescents, indicating that behavioral and objective measures of social response may not always be aligned. This is an important consideration when using objective measures of neural activity and emphasizes the need to examine individual variables in addition to group differences. It is important to keep in mind that one of the criteria for participation in PEERS is that teens with ASD be motivated to make and keep friends; as such, teens in the current study were distinctly socially motivated. Consequently, future studies measuring neural changes before and after intervention in adolescents and/or adults with ASD should consider participant motivation, as it is often required in these groups.

Although between-group differences were not observed, within-group variability of adolescents with ASD shed light on individual differences that affect social reward responsivity after intervention. Individual change in neural correlates of response to social reward was predicted by parent-reported social motivation before intervention and active engagement during the program. Participants who were *more* actively engaged in PEERS and who displayed *more* social motivation prior to the start of intervention made the biggest gains in neural response to social rewards from pre- to post-intervention. Findings related to teen participation during intervention underscore the importance of engagement during behavioral intervention.

The effect of parent-reported social motivation prior to PEERS on changes in brain activity related to reward processing is the opposite of what we observed for social reward anticipation. That is, adolescents who had *lower* levels of parent-reported social motivation prior to PEERS displayed *greater* increases in neural correlates of social anticipation after PEERS, yet adolescents who had *higher* levels of parent-reported social motivation before PEERS displayed *increased* neural correlates of social reward responsivity after PEERS. This underscores the importance of dissociating social reward anticipation from social reward processing when considering individual response to intervention, as these constructs likely represent different neural processes. It may be that there are differences in saliency between wanting/anticipating social rewards vs. liking/responding to social rewards (55, 56) within the brain's reward system in individuals with ASD. These distinct cognitive processes offer a unique understanding of the Social Motivation Theory in adolescents with ASD who are driven to make and keep friends, suggesting that both motivation and reward systems may moderate intervention effects.

### Exploratory N170 Findings

Exploratory analyses were performed on the N170. A more robust N170 response approached significance at post-intervention compared to pre-intervention in the ASD group within the right hemisphere. This indicates an enhancement of facial processing after intervention that mirrors findings in neurotypical populations (32). It is important to note that the stimuli and ERP paradigm used in the current investigation were not designed to elicit N170 responses and thus differ from traditional measurements of the N170 (e.g., facial stimuli were positive in valence and contained additional reward-related information). Thus, findings from the N170 should be interpreted with caution.

### Limitations

Some limitations must be considered when interpreting results. Our sample size is small, and thus may have been underpowered to detect between-group differences. Inclusion of an ASD wait-list control group would have improved the experimental design of the investigation and may have allowed for the effects of the “natural passage of time” vs. “intervention” to be disentangled in the ASD group. However, inclusion of a TD group established, in-part, that change was not solely due to the passage of time. Change scores were used in this investigation instead of alternative methods of pre- and post-test analyses, which may have influenced results. A clustered design was not utilized in this design and this may have impacted our statistical power and effect size of intervention effects (57). Additionally, a small sample size reduces our ability to generalize our findings to larger groups of adolescents with ASD. Given the cognitive demands of PEERS and the EEG procedures, participants were required to have cognitive abilities in the average range to be eligible for the current study (i.e., IQ ≤ 70). Another requirement was for teens with ASD to be motivated to make and keep friends and for both parents and teens to be able to attend weekly 90-min intervention sessions for 16 weeks. Given these considerations, it is likely that participants in the current study represent a subset of adolescents with ASD. In the future, it will be important to clarify which of these factors may affect the efficacy of PEERS.

### Conclusion

To our knowledge, this is the first study to measure neural correlates of both social reward anticipation and processing in adolescents with ASD before and after the PEERS intervention. Findings supported our hypothesis that change in neural correlates of social reward anticipation and processing can be predicted by individual characteristics prior to intervention. Although traditional conceptualizations of social motivation define this construct as the desire or intention to engage and interact with others, our findings reinforce previous work that reward anticipation and reward processing are dissociable constructs (56, 58). Our findings suggest that for individuals with ASD who may have lower levels of intrinsic motivation to interact with others, PEERS may enhance their desire to approach others, commonly known as approach motivation, or “wanting” to interact (as indicated by increased neural reward anticipation to faces; SPN). However, for those who are already motivated to interact with others, completion of the PEERS program may further reinforce social interactions as pleasant (as indicated by increased neural reward processing of faces, RewP).

In ASD intervention research, there remains a lack of validated biomarkers that can be used to predict intervention outcomes (59). Future studies with larger samples should attempt to both replicate these findings and further parse these constructs to move closer to “precision medicine” efforts to individualize intervention and predict which adolescents are most likely to benefit from PEERS.

## Data Availability Statement

The raw data supporting the conclusions of this article will be made available by the authors, without undue reservation.

## Ethics Statement

The studies involving human participants were reviewed and approved by Institutional Review Board at the University of California, Riverside. Written informed consent to participate in this study was provided by the participants' legal guardian/next of kin.

## Author Contributions

KS designed the experiment. EB and KS conceptualized the analysis strategy. EB performed the EEG processing, statistical analysis, statistical interpretation, and drafting of the manuscript under the supervision of KS. JB verified the analytical methods and interpretations. EV and TC reviewed and confirmed descriptions of methodology. All authors discussed the results, contributed to the final published manuscript, and have read and agreed to the published version of the manuscript.

## Funding

Support for this work was provided by the Bezos Family Grant Autism, the Social Brain, and Neuroscience: Treating Underserved Latino Teenagers in the Inland Empire.

## Conflict of Interest

The authors declare that the research was conducted in the absence of any commercial or financial relationships that could be construed as a potential conflict of interest. The reviewer JW declared a shared affiliation, with no collaboration, with the authors EB, EV, TC, JB, and KS at the time of the review.

## Publisher's Note

All claims expressed in this article are solely those of the authors and do not necessarily represent those of their affiliated organizations, or those of the publisher, the editors and the reviewers. Any product that may be evaluated in this article, or claim that may be made by its manufacturer, is not guaranteed or endorsed by the publisher.

## References

[1] Kenny L Hattersley C Molins B Buckley C Povey C Pellicano E. Which terms should be used to describe autism? Perspectives from the UK autism community. Autism. (2016) 20:442–62. 10.1177/1362361315588200 26134030

[2] Jones W Carr K Klin A. Absence of preferential looking to the eyes of approaching adults predicts level of social disability in 2-year-old toddlers with autism spectrum disorder. Arch Gen Psychiatry. (2008) 65:946–54. 10.1001/archpsyc.65.8.946 18678799

[3] Pierce K Marinero S Hazin R McKenna B Barnes CC Malige A. Eye tracking reveals abnormal visual preference for geometric images as an early biomarker of an autism spectrum disorder subtype associated with increased symptom severity. Biol Psychiatry. (2016) 79:657–66. 10.1016/j.biopsych.2015.03.032 25981170 PMC4600640

[4] Chevallier C Kohls G Troiani V Brodkin ES Schultz RT. The social motivation theory of autism. Trends Cogn Sci. (2012) 16:231–9. 10.1016/j.tics.2012.02.007 22425667 PMC3329932

[5] Bottini S. Social reward processing in individuals with autism spectrum disorder: a systematic review of the social motivation hypothesis. Res Autism Spectr Disord. (2018) 45:9–26. 10.1016/j.rasd.2017.10.00129898209

[6] Haber SN Knutson B. The reward circuit: linking primate anatomy and human imaging. Neuropsychopharmacol. (2010) 35:4–26. 10.1038/npp.2009.129 19812543 PMC3055449

[7] Supekar K Kochalka J Schaer M Wakeman H Qin S Padmanabhan A. Deficits in mesolimbic reward pathway underlie social interaction impairments in children with autism. Brain. (2018) 141:2795–805. 10.1093/brain/awy191 30016410 PMC6113649

[8] Kohls G Antezana L Mosner MG Schultz RT Yerys BE. Altered reward system reactivity for personalized circumscribed interests in autism. Mol Autism. (2018) 9:9. 10.1186/s13229-018-0195-7 29423135 PMC5791309

[9] Stavropoulos KKM Carver LJ. Reward anticipation and processing of social versus nonsocial stimuli in children with and without autism spectrum disorders. J Child Psychol Psychiatry. (2014) 55:1398–408. 10.1111/jcpp.12270 24890037

[10] Dichter GS Richey JA Rittenberg AM Sabatino A Bodfish JW. Reward circuitry function in autism during face anticipation and outcomes. J Autism Dev Disord. (2012) 42:147–60. 10.1007/s10803-011-1221-1 22187105 PMC8624275

[11] Meyer GM Marco-Pallarés J Boulinguez P Sescousse G. Electrophysiological underpinnings of reward processing: ARE we exploiting the full potential of EEG? Neuroimage. (2021) 242:118478. 10.1016/j.neuroimage.2021.118478 34403744

[12] Clements CC Zoltowski AR Yankowitz LD Yerys BE Schultz RT Herrington JD. Evaluation of the social motivation hypothesis of autism: a systematic review and meta-analysis. JAMA Psychiatry. (2018) 75:797. 10.1001/jamapsychiatry.2018.1100 29898209 PMC6143096

[13] Holroyd CB Pakzad-Vaezi KL Krigolson OE. The feedback correct-related positivity: sensitivity of the event-related brain potential to unexpected positive feedback. Psychophysiology. (2008) 45:688–97. 10.1111/j.1469-8986.2008.00668.x 18513364

[14] Proudfit GH. The reward positivity: from basic research on reward to a biomarker for depression. Psychophysiology. (2015) 52:449–59. 10.1111/psyp.12370 25327938

[15] Stavropoulos KKM Carver LJ. Effect of familiarity on reward anticipation in children with and without autism spectrum disorders. PLoS ONE. (2014) 9:e106667. 10.1371/journal.pone.0106667 25184524 PMC4153666

[16] Baker E Veytsman E Martin AM Blacher J Stavropoulos KKM. Increased neural reward responsivity in adolescents with ASD after social skills intervention. Brain Sci. (2020) 10:402. 10.3390/brainsci10060402 32599849 PMC7349909

[17] Dawson G Burner K. Behavioral interventions in children and adolescents with autism spectrum disorder: a review of recent findings. Curr Opin Pediatr. (2011) 23:616–20. 10.1097/MOP.0b013e32834cf082 22037220

[18] Reichow B Boyd BA Barton EE Odom SL. Handbook of Early Childhood Special Education. New York, NY: Springer Science+Business Media (2016).

[19] Laugeson EA. The PEERS Curriculum for School-Based Professionals. New York, NY: Routledge (2013) 480p. 10.4324/9780203102374

[20] Laugeson EA Frankel F Gantman A Dillon AR Mogil C. Evidence-based social skills training for adolescents with autism spectrum disorders: the UCLA PEERS program. J Autism Dev Disord. (2012) 42:1025–36. 10.1007/s10803-011-1339-1 21858588

[21] Laugeson EA Frankel F. Social Skills for Teenagers With Developmental and Autism Spectrum Disorders: The PEERS Treatment Manual. New York, NY: Routledge (2011) 446p. 10.4324/9780203867686 25860311

[22] Laugeson EA Frankel F Mogil C Dillon AR. Parent-assisted social skills training to improve friendships in teens with autism spectrum disorders. J Autism Dev Disord. (2009) 39:596–606. 10.1007/s10803-008-0664-5 19015968

[23] Dawson G Jones EJH Merkle K Venema K Lowy R Faja S. Early behavioral intervention is associated with normalized brain activity in young children with autism. J Am Acad Child Adolesc Psychiatry. (2012) 51:1150–9. 10.1016/j.jaac.2012.08.018 23101741 PMC3607427

[24] Van Hecke AV Stevens S Carson AM Karst JS Dolan B Schohl K. Measuring the plasticity of social approach: a randomized controlled trial of the effects of the PEERS intervention on EEG asymmetry in adolescents with autism spectrum disorders. J Autism Dev Disord. (2015) 45:316–35. 10.1007/s10803-013-1883-y 23812665

[25] Venkataraman A Yang DYJ Dvornek N Staib LH Duncan JS Pelphrey KA. Pivotal response treatment prompts a functional rewiring of the brain among individuals with autism spectrum disorder. Neuroreport. (2016) 27:1081–5. 10.1097/WNR.0000000000000662 27532879 PMC5007196

[26] Ventola P Yang DYJ Friedman HE Oosting D Wolf J Sukhodolsky DG. Heterogeneity of neural mechanisms of response to pivotal response treatment. Brain Imaging Behav. (2015) 9:74–88. 10.1007/s11682-014-9331-y 25370452 PMC4993028

[27] Voos AC Pelphrey KA Tirrell J Bolling DZ Vander Wyk B Kaiser MD. Neural mechanisms of improvements in social motivation after pivotal response treatment: two case studies. J Autism Dev Disord. (2013) 43:1–10. 10.1007/s10803-012-1683-9 23104615 PMC4999079

[28] Yang D Pelphrey KA Sukhodolsky DG Crowley MJ Dayan E Dvornek NC. Brain responses to biological motion predict treatment outcome in young children with autism. Transl Psychiatry. (2016) 15:e948. 10.1038/tp.2016.213 27845779 PMC5314125

[29] Kala S Rolison MJ Trevisan DA Naples AJ Pelphrey K Ventola P. Brief report: preliminary evidence of the N170 as a biomarker of response to treatment in autism spectrum disorder. Front Psychiatry. (2021) 12:709382. 10.3389/fpsyt.2021.709382 34267691 PMC8275957

[30] Key AP Corbett BA. The unfulfilled promise of the N170 as a social biomarker. Biol Psychiatry Cogn Neurosci Neuroimaging. (2020) 5:342–53. 10.1016/j.bpsc.2019.08.011 31679960 PMC7064396

[31] McPartland JC Bernier RA Jeste SS Dawson G Nelson CA Chawarska K. The autism biomarkers consortium for clinical trials (ABC-CT): scientific context, study design, and progress toward biomarker qualification. Front Integr Neurosci. (2020) 14:16. 10.3389/fnint.2020.00016 32346363 PMC7173348

[32] Kang E Keifer CM Levy EJ Foss-Feig JH McPartland JC Lerner MD. Atypicality of the N170 event-related potential in autism spectrum disorder: a meta-analysis. Biol Psychiatry Cogn Neurosci Neuroimaging. (2018) 3:657–66. 10.1016/j.bpsc.2017.11.003 30092916 PMC6089230

[33] McPartland J Dawson G Webb SJ Panagiotides H Carver LJ. Event-related brain potentials reveal anomalies in temporal processing of faces in autism spectrum disorder. J Child Psychol Psychiatry. (2004) 45:1235–45. 10.1111/j.1469-7610.2004.00318.x 15335344

[34] Webb SJ Dawson G Bernier R Panagiotides H. ERP evidence of atypical face processing in young children with autism. J Autism Dev Disord. (2006) 36:881–90. 10.1007/s10803-006-0126-x 16897400 PMC2989721

[35] O'Connor K Hamm JP Kirk IJ. The neurophysiological correlates of face processing in adults and children with Asperger's syndrome. Brain Cogn. (2005) 59:82–95. 10.1016/j.bandc.2005.05.004 16009478

[36] Miskovic V Schmidt LA Georgiades K Boyle M Macmillan HL. Adolescent females exposed to child maltreatment exhibit atypical EEG coherence and psychiatric impairment: linking early adversity, the brain, and psychopathology. Dev Psychopathol. (2010) 22:419–32. 10.1017/S0954579410000155 20423551

[37] Mitchell AM Pössel P. Frontal brain activity pattern predicts depression in adolescent boys. Biol Psychol. (2012) 89:525–7. 10.1016/j.biopsycho.2011.12.008 22200655

[38] Lord C Rutter M DiLavore PC Risi S Gotham K Bishop SL. Autism Diagnostic Observation Schedule. 2nd ed. Torrance, CA: WPS (2012).

[39] Wechsler D. Wechsler Abbreviated Scale of Intelligence. 2nd ed. San Antonio, TX: Pearson (2011).

[40] Constantino JN. Social Responsiveness Scale. 2nd ed. Torrance, CA: WPS (2012).

[41] Gresham FM Elliott SN. Social Skills Improvement System Rating Scales. Minneapolis, MN: Pearson (2008).

[42] Laugeson EA Gantman A Kapp SK Orenski K Ellingsen R. A randomized controlled trial to improve social skills in young adults with autism spectrum disorder: the UCLA PEERS program. J Autism Dev Disord. (2015) 45:3978–89. 10.1007/s10803-015-2504-8 26109247

[43] Ellingsen R Bolton C Laugeson E. Evidence-based social skills groups for individuals with autism spectrum disorder across the lifespan. In: Leaf JB, editor. Handbook of Social Skills and Autism Spectrum Disorder. Cham: Springer International Publishing (2017). 10.1007/978-3-319-62995-7_20

[44] Tottenham N Tanaka JW Leon AC McCarry T Nurse M Hare TA. The NimStim set of facial expressions: judgments from untrained research participants. Psychiatry Res. (2009) 168:242–9. 10.1016/j.psychres.2008.05.006 19564050 PMC3474329

[45] Lopez-Calderon J Luck SJ. ERPLAB: an open-source toolbox for the analysis of event-related potentials. Front Hum Neurosci. (2014) 8:213. 10.3389/fnhum.2014.00213 24782741 PMC3995046

[46] Hajcak G Moser JS Yeung N Simons RF. On the ERN and the significance of errors. Psychophysiology. (2005) 42:151–60. 10.1111/j.1469-8986.2005.00270.x 15787852

[47] Hajcak G Moser JS Holroyd CB Simons RF. The feedback-related negativity reflects the binary evaluation of good versus bad outcomes. Biol Psychol. (2006) 71:148–54. 10.1016/j.biopsycho.2005.04.001 16005561

[48] Blau VC Maurer U Tottenham N McCandliss BD. The face-specific N170 component is modulated by emotional facial expression. Behav Brain Funct. (2007) 3:7. 10.1186/1744-9081-3-7 17244356 PMC1794418

[49] Dawson G Webb SJ McPartland J. Understanding the nature of face processing impairment in autism: insights from behavioral and electrophysiological studies. Dev Neuropsychol. (2005) 27:403–24. 10.1207/s15326942dn2703_6 15843104

[50] Allison PD. Change scores as dependent variables in regression analysis. Sociol Methodol. (1990) 20:93–114. 10.2307/271083

[51] Green SB. How many subjects does it take to do a regression analysis. Multivariate Behav Res. (1991) 26:499–510. 10.1207/s15327906mbr2603_7 26776715

[52] Tabachnick BG Fidell LS. Using Multivariate Statistics. Harper & Row (1989) 774p.

[53] Stavropoulos KKM Carver LJ. Reward sensitivity to faces versus objects in children: an ERP study. Soc Cogn Affect Neurosci. (2014) 9:1569–75. 10.1093/scan/nst149 24036961 PMC4187274

[54] Scott-Van Zeeland AA Dapretto M Ghahremani DG Poldrack RA Bookheimer SY. Reward processing in autism. Autism Res. (2010) 3:53–67. 10.1002/aur.122 20437601 PMC3076289

[55] Kohls G Schulte-Rüther M Nehrkorn B Müller K Fink GR Kamp-Becker I. Reward system dysfunction in autism spectrum disorders. Soc Cogn Affect Neurosci. (2013) 8:565–72. 10.1093/scan/nss033 22419119 PMC3682440

[56] Berridge KC Robinson TE Aldridge JW. Dissecting components of reward: ‘liking’, ‘wanting’, and learning. Curr Opin Pharmacol. (2009) 9:65–73. 10.1016/j.coph.2008.12.014 19162544 PMC2756052

[57] Lopata C Donnelly JP Rodgers JD Thomeer ML. Systematic review of data analyses and reporting in group-based social skills intervention RCTs for youth with ASD. Res Autism Spectr Disord. (2019) 59:10–21. 10.1016/j.rasd.2018.11.008

[58] Kohls G Chevallier C Troiani V Schultz RT. Social ‘wanting’ dysfunction in autism: neurobiological underpinnings and treatment implications. J Neurodev Disord. (2012) 4:10. 10.1186/1866-1955-4-10 22958468 PMC3436671

[59] McCracken JT Anagnostou E Arango C Dawson G Farchione T Mantua V. Drug development for autism spectrum disorder (ASD): progress, challenges, and future directions. Euro Neuropsychopharmacol. (2021) 48:3–31. 10.1016/j.euroneuro.2021.05.010 34158222 PMC10062405

